# Supplementary material for: Use of copula to model within‐study association in bivariate meta‐analysis of binomial data at the aggregate level: A Bayesian approach and application to surrogate endpoint evaluation
Source: Stat Med. 2022 Aug 5;41(25):4961–81. doi: 10.1002/sim.9547 (PMC9804662; doi:10.1002/sim.9547)
Supplement: Supplementary file 1 — Data S1 Supplementary material [file SIM-41-4961-s001.pdf]

Supplementary material

A

A.1 Boostrap method to estimate the within-study correlation of BRMA

A bootstrap method was used to estimate the within-study correlations ρ_{wi} for each study i between the treatment effects on both outcomes by drawing 3000 bootstrap samples with replacement from the IPD. The treatment effects (logOR) on each outcome and were calculated for each bootstrap sample by using the standard formulas and the Pearson's correlation coefficient between the treatment effects (logOR) were obtained.

```
bootstrap1 = function(df,Nb){
#Nb=number of bootstrap samples, df= dataframe containing IPD
names(df) = paste(c('Y1A','Y2A','Y1B','Y2B'))
s         = length(df$Y1A)#number of observations in the data
y1=y2=array(0,Nb)
for (d in 1:Nb){
sam       = sample(s, replace=T)
boot.1    = df$Y1A[sam]
boot.2    = df$Y1B[sam]
boot.3    = df$Y2A[sam]
boot.4    = df$Y2B[sam]
r1A       = sum(boot.1)
r1B       = sum(boot.2)
r2A       = sum(boot.3)
r2B       = sum(boot.4)
#Log odds ratio on the first outcome
LOR1      = log(((r1B+0.5)*(s-r1A+0.5))/((s-r1B+0.5)*(r1A+0.5)))
#Log odds ratio on the second outcome
LOR2      = log(((r2B+0.5)*(s-r2A+0.5))/((s-r2B+0.5)*(r2A+0.5)))
y1[d]     = LOR1
y2[d]     = LOR2}
#the correlations between log odds ratios across bootstrap samples
rho = cor(y1,y2,method= "pearson")
return(list(rho=rho)) }
```

A.2 Bootstrap method to estimate the within-study association parameter of BRMA-BC

Another bootstrap method was used to estimate the dependence parameters ρ_A, ρ_B of the joint density made with the normal copula. Similarly as in the previous bootstrap method 3000 bootstrap samples with replacement were drawn from IPD. Summary data were calculated for each bootstrap sample and then dependence parameters of the bivariate normal copula were estimated by using a optimiser such as *nlminb* in R.

```
bootstrap2 = function (df,Nb){
  names(df)       = paste(c('Y1A','Y2A','Y1B','Y2B'))
  s               <-length(df$Y1A)   #number of observations in the data
  y1A<-y1B<-y2A<-y2B<-array(1,Nb)
  for (k in 1:Nb){
   while ((y1A[k]==1 &&y2A[k]==1) |(y1B[k]==1 &&y2B[k]==1) ){
   sam<-sample(s, replace=T)
   boot.1   <-df$Y1A[sam]
   boot.2   <-df$Y1B[sam]
   boot.3   <-df$Y2A[sam]
   boot.4   <-df$Y2B[sam]
   y1A[k]   <-sum(boot.1)
   y1B[k]   <-sum(boot.2)
   y2A[k]   <-sum(boot.3)
   y2B[k]   <-sum(boot.4)}
  }
  llik1 <- function(p)-sum(dbinom(y1A,prob=p,size=s,log=TRUE))
  llik2 <- function(p)-sum(dbinom(y1B,prob=p,size=s,log=TRUE))
  llik3 <- function(p)-sum(dbinom(y2A,prob=p,size=s,log=TRUE))
  llik4 <- function(p)-sum(dbinom(y2B,prob=p,size=s,log=TRUE))
  p1A.hat     = optimize(llik1, c(0,1))$min
  p1B.hat     = optimize(llik2, c(0,1))$min
  p2A.hat     = optimize(llik3, c(0,1))$min
  p2B.hat     = optimize(llik4, c(0,1))$min
  uA          = pbinom(y1A,s,p1A.hat)
  uA1         = pbinom(y1A-1,s,p1A.hat)
  vA          = pbinom(y2A,s,p2A.hat)
  vA1         = pbinom(y2A-1,s,p2A.hat)
  uB          = pbinom(y1B,s,p1B.hat)
  uB1         = pbinom(y1B-1,s,p1B.hat)
  vB          = pbinom(y2B,s,p2B.hat)
  vB1         = pbinom(y2B-1,s,p2B.hat)
  fA = function(theta1) {-sum(log(cop.pmf2(theta1,uA,vA,uA1,vA1)))}
  fB = function(theta2) {-sum(log(cop.pmf2(theta2,uB,vB,uB1,vB1)))}
  optf1  = nlminb(c(.2),fA,lower = 1.00001,upper=9,
```

```
        control = list(iter.max=1000,eval.max=1000))
    optf2  = nlminb(c(.2),fB,lower = 1.00001,upper=9,
        control = list(iter.max=1000,eval.max=1000))
    return(list(thetaf1=optf1$par,thetaf2=optf2$par,
    diagnostic_1 = optf1$convergence, diagnostic_2 = optf2$convergence))}


#cdf of bivariate normal copula
pbvncop=function(u,v,cpar)
{ # endpoint corrections to prevent NaN
u[1-u<1.e-9]=1-1.e-9
v[1-v<1.e-9]=1-1.e-9
u[u<1.e-9]=1.e-9
v[v<1.e-9]=1.e-9
t <- qnorm(u)
s <- qnorm(v)
cdf=pbivnorm(t,s,cpar)
cdf
}


    # joint pmf funnction

cop.pmf2 = function(theta,u,v,u1,v1) {
pbvncop(u,v,theta)-pbvncop(u1,v,theta)-pbvncop(u,v1,theta)+pbvncop(u1,v1,theta)
}
```

A.3 STAN code of BRMA

```
data{
int<lower = 0> Ns;
int<lower = 0> nA[Ns,2];
int<lower = 0> nB[Ns,2];
int<lower = 0> rA[Ns,2];
int<lower = 0> rB[Ns,2];
real rho_w[Ns];}

transformed data{
//Calculate log odds ratios
vector[2] Y[Ns];
```

```
vector[2] S[Ns];
for (i in 1:Ns){
Y[i,1]=log(((rB[i,1]+0.5)*(nA[i,1]-rA[i,1]+0.5))/((nB[i,1]-rB[i,1]+0.5)*(rA[i,1]+0.5)));
Y[i,2]=log(((rB[i,2]+0.5)*(nA[i,2]-rA[i,2]+0.5))/((nB[i,2]-rB[i,2]+0.5)*(rA[i,2]+0.5)));
S[i,1]=sqrt((1/(rB[i,1]+0.5))+(1/(nB[i,1]-rB[i,1]+0.5))+
(1/(rA[i,1]+0.5))+(1/(nA[i,1]-rA[i,1]+0.5)));
S[i,2]=sqrt((1/(rB[i,2]+0.5))+(1/(nB[i,2]-rB[i,2]+0.5))+
(1/(rA[i,2]+0.5))+(1/(nA[i,2]-rA[i,2]+0.5)));}}

parameters{
real rr;
vector[2]  b;
vector[2]  z[Ns];
vector<lower=0, upper=5>[2] tau;}

transformed parameters{
matrix[2,2] Tau;
matrix[2,2] L;
matrix[2,2] Sigma1[Ns];
vector[2] delta[Ns];
real<lower= -1, upper=1> rho1;
rho1           = tanh(rr);
for (i in 1:Ns){
Sigma1[i,1, 1] = S[i,1]^2;
Sigma1[i,1, 2] = S[i,1]*S[i,2]*rho_w[Ns];
Sigma1[i,2, 1] = S[i,1]*S[i,2]*rho_w[Ns];
Sigma1[i,2, 2] = S[i,2]^2;}
Tau[1,1]       = tau[1]^2;
Tau[2,2]       = tau[2]^2;
Tau[1,2]       = tau[1]*tau[2]*rho1;
Tau[2,1]       = tau[1]*tau[2]*rho1;
L              = cholesky_decompose(Tau);
//non-centred parameterisation for delta~multi_normal(b,Tau)
for (i in 1:Ns){
delta[i] = b + (L*z[i]);}

model{
//priors
rr       ~ std_normal();
b        ~ normal(0, 10);
for (i in 1:Ns){
```

```
z[i]      ~ std_normal();
//likelihood
Y[i]      ~ multi_normal(delta[i],Sigma1[i]);}}
```

A.4 STAN code of BRMA-IB

```
data{
int<lower = 0> Ns;
int<lower = 0> nA[Ns,2];
int<lower = 0> nB[Ns,2];
int<lower = 0> rA[Ns,2];
int<lower = 0> rB[Ns,2];}

parameters {
real rr;
vector[2] b;
vector<lower = 0, upper = 5>[2] tau;
vector[2] z[Ns];
vector[2] mu[Ns];}

transformed parameters{
matrix[2,2] Tau;
matrix[2,2] L;
vector[2] delta[Ns];
real<lower= -1, upper=1> rho1;
rho1      = tanh(rr);
Tau[1, 1] = tau[1]^2;
Tau[1, 2] = tau[1]*tau[2]*rho1;
Tau[2, 1] = tau[1]*tau[2]*rho1;
Tau[2, 2] = tau[2]^2;
L         = cholesky_decompose(Tau);
//non-centred parameterisation for delta~multi_normal(b,Tau)
for (i in 1:Ns){
delta[i]  = b+ L*z[i];}}

model {
//priors
b        ~ normal(0, 10);
rr       ~ std_normal();
for (i in 1:Ns){
z[i]     ~ std_normal();
mu[i]    ~ normal(0, 10);
//likelihoods
rA[i,1]  ~ binomial_logit(nA[i,1], mu[i,1]);
rA[i,2]  ~ binomial_logit(nA[i,2], mu[i,2]);
```

```
rB[i,1]  ~ binomial_logit(nB[i,1], mu[i,1]+delta[i,1]);
rB[i,2]  ~ binomial_logit(nB[i,2], mu[i,2]+delta[i,2]); }}
```

A.5 STAN code of BRMA-BC

```
functions {
//Normal Copula CDF
//This is devoloped by stan team:
//https://mc-stan.org/docs/2_28/stan-users-guide/examples.html
real fcop3(real theta, real u1, real u2){
real z1 = inv_Phi(u1);
real z2 = inv_Phi(u2);
if (z1 != 0 || z2 != 0) {
real denom = fabs(theta) < 1.0 ? sqrt((1 + theta)*(1 - theta)) : not_a_number();
real a1 = (z2 / z1 - theta) / denom;
real a2 = (z1 / z2 - theta) / denom;
real product = z1 * z2;
real delta = product < 0 || (product == 0 && (z1 + z2) < 0);
return 0.5 * (u1 + u2 - delta) - owens_t(z1, a1) - owens_t(z2, a2);
}
return 0.25 + asin(theta) / (2 * pi());
}

real Bivfcop_lpmf(int[] r,int[] n, real theta, vector p){
vector[2] f;
vector[2] f1;
real prob;
for(i in 1:2){
f1[i] = binomial_cdf(r[i] - 1| n[i], p[i]);
if (f1[i] > 1-1.e-9) f1[i] = 1-1.e-9;
if (f1[i] < 1.e-9) f1[i] = 1.e-9;
f[i]  = binomial_cdf(r[i] | n[i], p[i]);
if (f[i] > 1-1.e-9) f[i] = 1-1.e-9;
if (f[i] < 1.e-9) f[i] = 1.e-9;}
prob = fcop3(theta,f[1],f[2])-fcop3(theta,f[1],f1[2])-fcop3(theta,f1[1],f[2])+
fcop3(theta,f1[1],f1[2]);
return log(prob);}
}

data{
int<lower = 0> Ns;
int<lower = 0> nA[Ns,2];
int<lower = 0> nB[Ns,2];
int<lower = 0> rA[Ns,2];
```

```
int<lower = 0> rB[Ns,2];
real theta1[Ns];real theta2[Ns];}

parameters{
real rr;
vector[2] b;
vector<lower = 0, upper = 3>[2] tau;
vector[2] z[Ns];
vector[2] mu[Ns];
}

transformed parameters{
cholesky_factor_cov[2] L;
cov_matrix[2] Tau;
vector[2] delta[Ns];
vector<lower=0, upper=1>[2] pA[Ns];
vector<lower=0, upper=1>[2] pB[Ns];
real<lower= -1, upper=1> rho1;
rho1     = tanh(rr);
Tau[1,1] = tau[1]^2;
Tau[2,2] = tau[2]^2;
Tau[1,2] = tau[1]*tau[2]*rho1;
Tau[2,1] = tau[1]*tau[2]*rho1;
L        = cholesky_decompose(Tau);
for (i in 1:Ns){
delta[i] = b + (L*z[i]);
pA[i] = inv_logit(mu[i]);
pB[i] = inv_logit(mu[i]+delta[i]);}
}

model{
rr       ~ std_normal();
b        ~ normal(0, 10);
mu[,1]   ~ normal(0, 10);
mu[,2]   ~ normal(0, 10);
z[,1]    ~ std_normal();
z[,2]    ~ std_normal();
for (i in 1:Ns){
//likelihoods
//sample from the joint pmfs with binomial marginals
target += Bivfcop_lpmf(rA[i] | nA[i], theta1[i], pA[i]);
```

```
target += Bivfcop_lpmf(rB[i] | nB[i], theta2[i], pB[i]); }
}
```

A.6 Double bootstrap method accounting for uncertainty around he within-study correlation

To obtain prior distributions for the within-study correlation ρ_w between treatment effects on CCyR and EFS and for the dependence parameters ρ_A, ρ_B between CCyR and EFS from pseudo IPD, we used a double bootstrap method. We sampled 3000 bootstrap samples (first level bootstrap samples) from the pseudo IPD and from each of the bootstrap samples we sampled another 3000 bootstrap samples (second level bootstrap samples). In the second level, we estimated ρ_w and the dependence parameters as in A.1 and A.2. This resulted in a set of 2000 estimates for each parameter (ρ_w, ρ_A, ρ_B). We used these sets as empirical prior distributions for these parameters.

A.7 Dependence parameters of the generation process

Data of the simulation study were generated using the R package *copula*. To simulate IPD with low, moderate and high association, we used joint densities constructed with normal copula of Bernoulli marginal distributions in both arms with dependence parameters ρ_A, ρ_B. The following table presents the values of the dependence parameters for each scenario.

Table 1: Values of dependence parameters ρ_A and ρ_B for each set of scenarios corresponding to proportions of events (0.5 or 0.95)

Strength of association	Average Proportion of events = 0.5	Average Proportion of events = 0.95
Low within- study association	0.22	0.43
Moderate within-study association	0.60	0.76
Strong within-study association	0.91	0.95

A.8 Scenarios generated from independent Bernoulli distribution (no within-study association)

In this section we considered a set of scenarios with zero within-study association simulating IPD from independent Bernoulli distributions instead of copula dependent Bernoulli distributions. This allows us to assess the robustness of the estimates of BRMA-BC to different distributional assumptions of the data generation process.

Table 2 presents the empirical distribution of the within-study association parameters estimated with bootstrapping. It can be seen that the bootstrap methods estimated the within-study associations quite accurately and precisely across the three different sets of scenarios.

Table 3 presents the results of the estimates of between-studies correlation across the three sets of scenarios following the same format as in the simulation study. BRMA-BC performed better than BRMA and equally well as BRMA-IB in terms of RMSE, coverage probabilities and average bias in the scenarios with high proportions of events. This suggests that BRMA-BC gave robust estimates under a set of scenarios where no within-study association was present. Therefore we can conclude that it can efficiently model the within-study variability even when IPD are simulated under different distributional assumptions.

Table 2: Medians, 2.5% and 97.5% quantiles, of ρ_w, ρ_A and ρ_B estimated from the bootstrap samples across studies and 1000 simulation iterations when the number of patient in each study i was on average 300

Strength of association	Parameter	Average Proportion of events = 0.5	Average Proportion of events = 0.95
		Median 2.5% & 97.5%	Median 2.5% & 97.5%
zero	ρ_w	0.00 (-0.12, 0.12)	0.00 (-0.12, 0.12)
within-study	ρ_A	0.01 (-0.16, 0.16)	0.02 (-0.19, 0.21)
association	ρ_B	0.00 (-0.17, 0.16)	0.01 (-0.20, 0.19)

Table 3: Average bias of the estimates of ρ_b, coverage probabilities and RMSE of $\widehat{\rho_b}$ across 1000 simulations when within-study association was 0 and the average number of patients was 400.

Measure	Model	Average Proportion of events = 0.5	Average Proportion of events = 0.95
Average bias	BRMA	-0.01	-0.05
	BRMA-IB	-0.01	-0.04
	BRMA-BC	-0.01	-0.04
Coverage	BRMA	0.95	0.99
	BRMA-IB	0.95	0.98
	BRMA-BC	0.95	0.97
RMSE	BRMA	0.09	0.16
	BRMA-IB	0.09	0.12
	BRMA-BC	0.09	0.14

A.9 Convergence plots of the data example - CML

This section presents proof of convergence of the three models (Figures 1-6) for the CML data example. Convergence was reached for all the parameters of BRMA, BRMA-IB and BRMA-BC models as there is significant overlap between the density plots across chains and traceplots indicate good mixing of the chains as they look like a random scatter and significantly overlap across chains.

Figure 1: Trace plots (LHS plots) and density plots (RHS plots) of the between-studies parameters of BRMA

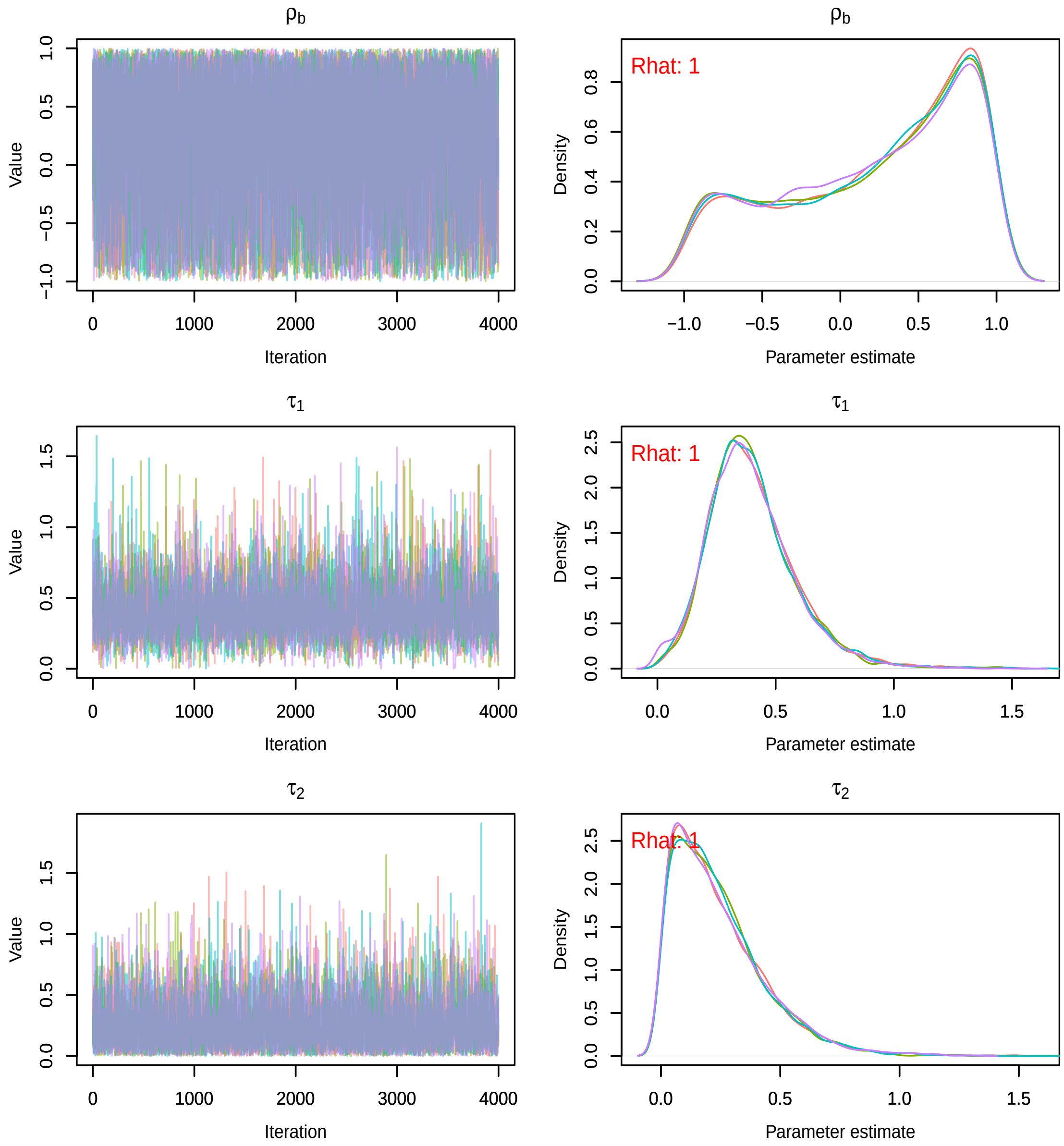

Figure 2: Trace plots (LHS plots) and density plots (RHS plots) of the between-studies parameters of BRMA

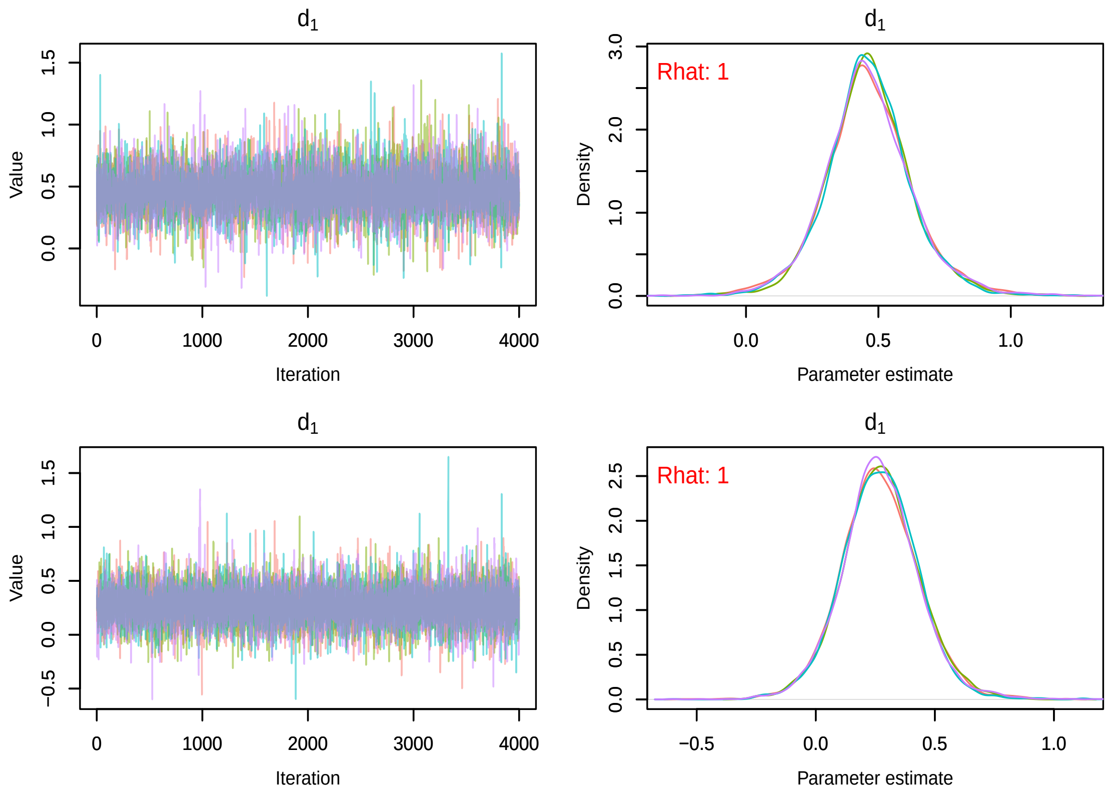

Figure 3: Trace plots (LHS plots) and density plots (RHS plots) of the between-studies parameters of BRMA-IB

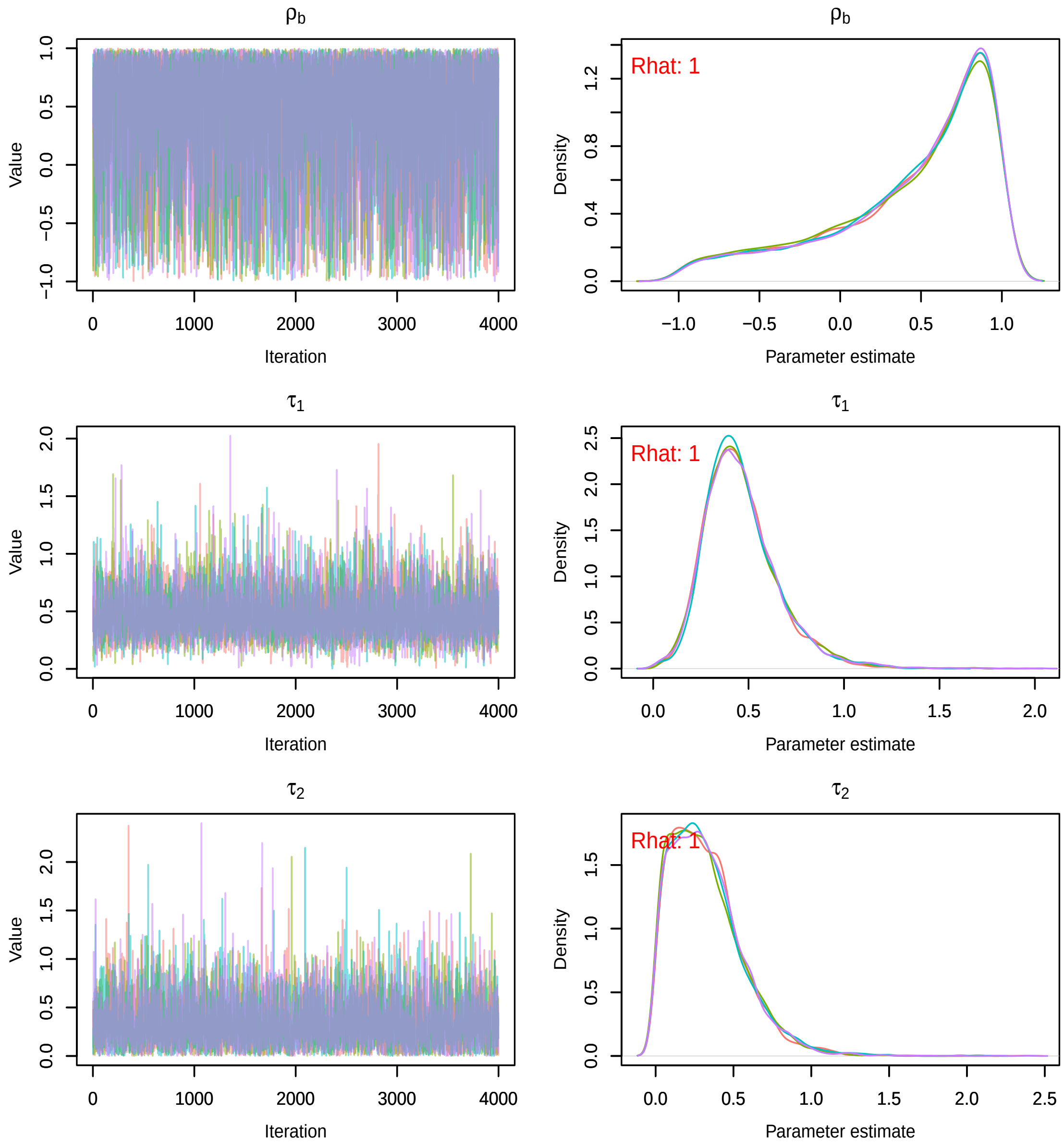

Figure 4: Trace plots (LHS plots) and density plots (RHS plots) of the between-studies parameters of BRMA-IB

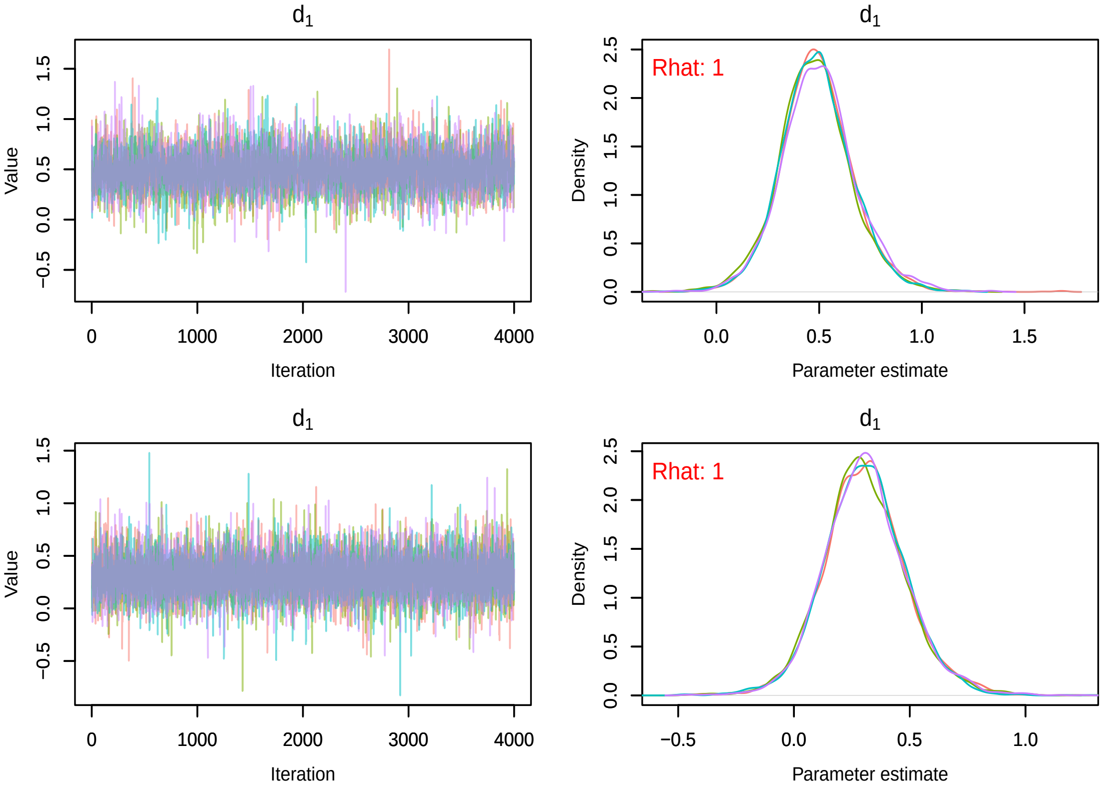

Figure 5: Trace plots (LHS plots) and density plots (RHS plots) of the between-studies parameters of BRMA-BC

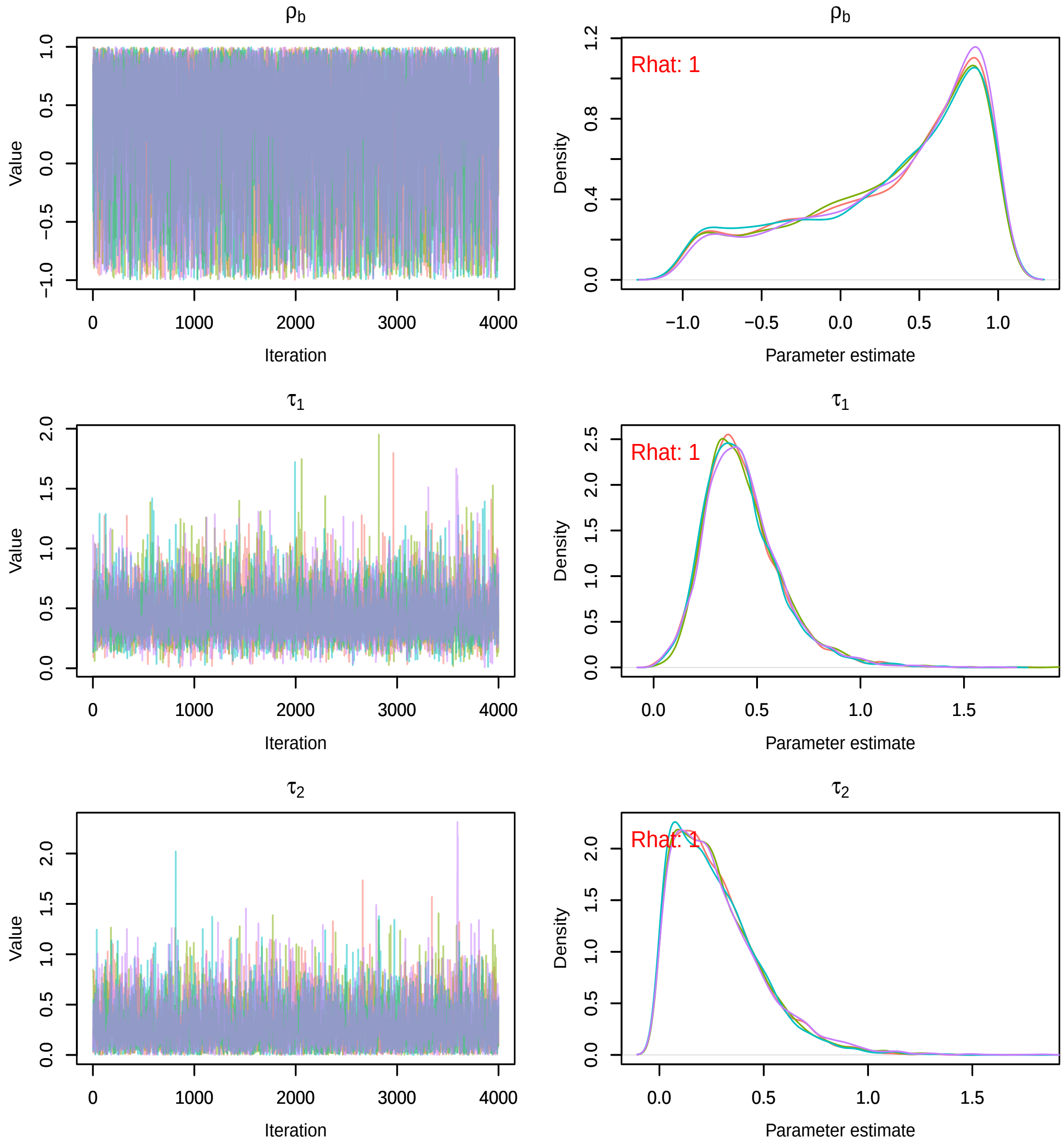

Figure 6: Trace plots (LHS plots) and density plots (RHS plots) of the between-studies parameters of BRMA-BC

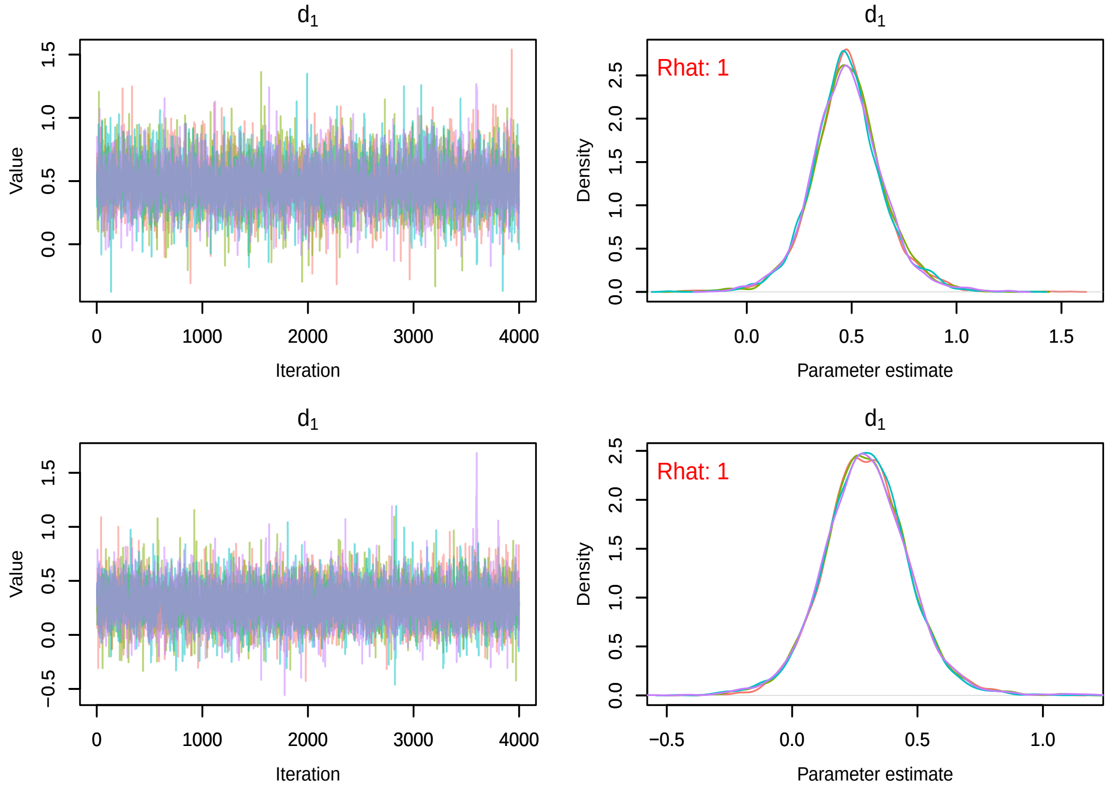

A.10 Convergence evaluation of the simulation study

This section discusses the convergence of the between-studies parameters of each model in the simulation study. Tables 4,5 and 6 provide information about the convergence of the parameters of each model for each scenario separately reporting the number of simulation iterations where $\widehat{R}$ exceeded 1.01. It can be seen that the total the number of iterations with $\widehat{R}$ >1.01 is very small for each model compared to the total number of simulation iterations (in total 1000 iterations were carried out). In such cases the particular this iteration was excluded for the Bayesian analysis.

The convergence status of the within-study association parameters ρ_A, ρ_B for each simulation iteration was evaluated by checking the convergence argument of the $nlminb$ optimiser in R. Whenever the optimiser failed to converge, IPD were re-simulated until the optimiser provided a reliable solution. Table 7 provides information about the number of simulation iterations where the optimiser returned a convergence error and IPD were re-simulated until the obtimiser was able to provide a converged solution for both of the dependence parameters. It can be seen that convergence problems were quite rare as the total the number of studies that IPD were re-simulated is very small compared to the total number of studies across all simulation iterations (in total 30 studies $\times$ 1000 simulation replication = 30000 studies were simulated). Across the simulation study the largest number was 258 studies i.e., it occurs approximately 8 times every 1000 studies.

Table 4: Number of simulation iteration with $\widehat{R}$ >1.01 for each scenario - BRMA model

Large study size

Strength of association	Parameter	Average Proportion of events = 0.5<br>Number of simulation iterations with $\widehat{R}$ >1.01	Average Proportion of events = 0.95<br>Number of simulation iterations with $\widehat{R} > 1.01$
Low within-study association	ρ_b	0	0
	τ_1	0	0
	τ_2	0	0
	d_1	0	0
	d_2	0	0
Moderate within-study association	ρ_b	0	0
	τ_1	0	0
	τ_2	0	0
	d_1	0	0
	d_2	0	0
High within-study association	ρ_b	0	0
	τ_1	0	0
	τ_2	0	0
	d_1	0	0
	d_2	0	0

Small study size

Strength of association	Parameter	Average Proportion of events = 0.5<br>Number of simulation iterations with $\widehat{R}$ >1.01	Average Proportion of events = 0.95<br>Number of simulation iterations with $\widehat{R} > 1.01$
Low within-study association	ρ_b	0	0
	τ_1	0	0
	τ_2	0	0
	d_1	0	0
	d_2	0	0
Moderate within-study association	ρ_b	0	2
	τ_1	0	0
	τ_2	0	0
	d_1	0	0
	d_2	0	0
High within-study association	ρ_b	1	7
	τ_1	0	0
	τ_2	0	0
	d_1	0	0
	d_2	0	0

Table 5: Number of simulation iteration with $\widehat{R}$ >1.01 for each scenario - BRMA-IB model

Large study size

		Average Proportion of events = 0.5	Average Proportion of events = 0.95
Strength of association	Parameter	Number of simulation iterations with $\widehat{R}$ >1.01	Number of simulation iterations with $\widehat{R} > 1.01$
Low within-study association	ρ_b	0	0
	τ_1	0	0
	τ_2	0	0
	d_1	0	0
	d_2	0	0
Moderate within-study association	ρ_b	0	0
	τ_1	0	0
	τ_2	0	0
	d_1	0	0
	d_2	0	0
High within-study association	ρ_b	0	0
	τ_1	0	0
	τ_2	0	0
	d_1	1	0
	d_2	0	0

Small study size

		Average Proportion of events = 0.5	Average Proportion of events = 0.95
Strength of association	Parameter	Number of simulation iterations with $\widehat{R}$ >1.01	Number of simulation iterations with $\widehat{R} > 1.01$
Low within-study association	ρ_b	0	0
	τ_1	0	0
	τ_2	0	0
	d_1	0	0
	d_2	0	0
Moderate within-study association	ρ_b	0	0
	τ_1	0	0
	τ_2	0	0
	d_1	0	0
	d_2	0	0
High within-study association	ρ_b	0	0
	τ_1	0	0
	τ_2	0	0
	d_1	0	0
	d_2	0	0

Table 6: Number of simulation iteration with $\widehat{R}$ >1.01 for each scenario - BRMA-BC model

Large study size

		Average Proportion of events = 0.5	Average Proportion of events = 0.95
Strength of association	Parameter	Number of simulation iterations with $\widehat{R}$ >1.01	Number of simulation iterations with $\widehat{R} > 1.01$
	ρ_b	0	0
Low within-study association	τ_1	0	0
	τ_2	0	0
	d_1	0	0
	d_2	0	0
	ρ_b	0	0
Moderate within-study association	τ_1	0	0
	τ_2	0	0
	d_1	0	0
	d_2	0	0
	ρ_b	0	0
High within-study association	τ_1	0	0
	τ_2	0	0
	d_1	0	0
	d_2	0	0

Small study size

		Average Proportion of events = 0.5	Average Proportion of events = 0.95
Strength of association	Parameter	Number of simulation iterations with $\widehat{R}$ >1.01	Number of simulation iterations with $\widehat{R} > 1.01$
	ρ_b	0	0
Low within-study association	τ_1	0	0
	τ_2	0	0
	d_1	0	0
	d_2	0	0
	ρ_b	0	2
Moderate within-study association	τ_1	0	0
	τ_2	0	0
	d_1	0	0
	d_2	0	0
	ρ_b	0	2
High within-study association	τ_1	0	0
	τ_2	0	0
	d_1	0	0
	d_2	0	0

Table 7: Number of simulation iterations where convergence was not initially reached either for ρ_A or ρ_B and IPD were re-simulated until convergence was reached.

Large study size	Average Proportion of events = 0.5	Average Proportion of events = 0.95
Strength of association	Number of simulation iterations	Number of simulation iterations
Low within-study association	42	101
Moderate within-study association	1	67
Strong within-study association	3	11

Small study size	Average Proportion of events = 0.5	Average Proportion of events = 0.95
Strength of association	Number of simulation iterations	Number of simulation iterations
Low within-study association	92	258
Moderate within-study association	10	228
Strong within-study association	2	40
